# Development, Alteration and Real Time Dynamics of Conjunctiva-Associated Lymphoid Tissue

**DOI:** 10.1371/journal.pone.0082355

**Published:** 2013-12-20

**Authors:** Sebastian Siebelmann, Uta Gehlsen, Gereon Hüttmann, Norbert Koop, Torsten Bölke, Andreas Gebert, Michael E. Stern, Jerry Y. Niederkorn, Philipp Steven

**Affiliations:** 1 Department of Ophthalmology, University of Cologne, Cologne, Germany; 2 Institute of Biomedical Optics, University of Lübeck, Lübeck, Germany; 3 Institute of Anatomy II, University Hospital Jena, Jena, Germany; 4 Biological Sciences, Allergan Inc., Irvine, California, United States of America; 5 Department of Ophthalmology, University of Texas Southwestern Medical Center, Dallas, Texas, United States of America; University of Leicester, United Kingdom of America

## Abstract

**Purpose:**

Conjunctiva-associated lymphoid tissue (CALT) is thought to play a key role in initiating ocular surface related immune responses. This study was planned to get first profound insights into the function of CALT related to development, cellular dynamics and morphological alteration using a novel mouse model.

**Methods:**

Expression and morphology of CALT were investigated using BALB/c mice kept under different housing conditions, after topical antigen-stimulation and following lymphadenectomy and splenectomy. Particles and bacteria were applied topically to study antigen-transport. Intravital visualization was performed using two-photon microscopy.

**Results:**

Postnatal development and ultrastructure of CALT in the mouse is similar to humans. Topical antigen-challenge significantly alters CALT expression. Bacterial translocation is demonstrated via lymphoepithelium whereas cellular velocities within follicles were approximately 8 µm/min.

**Conclusions:**

CALT in the mouse is an immunological interface of the ocular surface, featuring dynamic processes such as morphological plasticity, particle/bacteria transport and cellular migration.

## Introduction

The ocular surface represents a mucosal layer that despite its limited mechanical resistance facilitates a strong barrier against microbial and non-microbial pathogens. A constant interaction of pathogens with the host immune system and the related immunological activity is depicted by the presence of numerous immune cells such as B-cells, T-cells, macrophages and other antigen-presenting cells. These immune cells are not only located in a diffuse pattern throughout the conjunctiva but also as organized lymphoid follicles, namely the conjunctiva-associated lymphoid tissue (CALT) [1]. In coherence with well investigated organized lymphoid tissues of the intestine, CALT is thought to represent the immunological interface of the ocular surface with the external environment. It is hypothesized that CALT is in fact responsible for controlled antigen-uptake, -processing and –presentation, followed by initiation of an appropriate immune response and lymphocyte homing [2], [3]. Any reaction to foreign antigen would therefore be based on dynamic processes such as transport of antigen across barriers, cellular migration from, to and within different mucosal compartments and cell-cell interactions.

In humans CALT is frequently found in healthy eyes, demonstrating a physiological age-dependent time course with a lack of lymphoid follicles at birth, a peak in adolescence and steadily decrease as mice age [4], [5]. Inflammation of the ocular surface caused by chlamydia infection, allergy, dry-eye, viral and toxic conjunctivitis increases number and size of conjunctival lymphoid follicles, which can be easily detected in routine biomicroscopic examination [6]. These clinical findings together with descriptive histological investigations [7] implicate a functional role of CALT in ocular surface inflammation. However, functional studies of CALT are limited to the analysis of particle and antigen-transport across the lymphoepithelium in chicken, dogs, turkeys and rabbits [8]–[11], whereas other studies that verified any of the functional hypotheses stated above are not available until now. In summary, much is hypothesized but little is known about the function of CALT in general but also in the context of ocular surface diseases such as ocular allergy, infection or dry-eye.

In a previous study we introduced a mouse model that frequently contains CALT in the nictitating membrane of the eye following repeated topical stimulation with different antigens [12]. Using this model first functional immunological experiments became feasible in order to gain basic knowledge on the model used and first implications for its use in disease models. In this study we attempted to address the following hypotheses in order to gain insights into CALT function: i. Development of CALT in the mouse represents the human situation in terms of time dependence and expression of follicles ii. Animal housing condition and animal age influence CALT expression and may be crucial for designing experiments. iii. CALT is not constitutively expressed as are intestinal Peyer's patches [13] but is inducible by antigen-challenge similar to MALT such as bronchus-associated lymphoid tissue (BALT) [14] iv. CALT features cellular migration and cell exchange between different tissue compartments.

## Materials and Methods

### Ethics statement:

All experiments were conducted according to the Association for Research in Vision and Ophthalmology (ARVO) statement for the use of animals in Ophthalmic and Vision Research and with approval of the local animal committees of Schleswig-Holstein and Nordrhein-Westfalen (LANUV), Germany (Permit Numbers: 84-02.04.2011.A311; 95-8/09; 55-6/08). All surgery was performed under general anesthesia, and all efforts were made to minimize suffering.

### Animal experiments

Female BALB/c mice, aged 10 days to 24 weeks, were obtained from specific-pathogen free facilities at Charles River Laboratories (Sulzfeld, Germany) or the University of Kiel, Germany. Care and treatment of the animals were undertaken in accordance to the regulations of the Universities of Kiel and Cologne and performed either under short term anesthesia with Ketamine (Ketanest S®, Pfizer, Karlsruhe, Germany) and Xylazine (Rompun vet®, Bayer Health Care, Leverkusen, Germany) or long term anesthesia with Fentanyl (Bayer Health Care, Leverkusen, Germany), Midazolam Curamed (Actavis, Munich, Germany) and Medetomidine (Domitor, Pfizer, Berlin, Germany) intraperitoneal.

### Development of CALT

To study development of CALT mice aged 10 days, 4, 8, 10, 12, 16 and 24 weeks were euthanatized and whole eyes with adjacent nictitating membranes were dissected and fresh frozen in liquid nitrogen or a mixed solution of glutaraldehyde and formalin. Animals were kept under specific pathogen-free (SPF) conditions without topical antigen-challenge. To investigate the effect of housing-conditions on CALT, animals born under SPF conditions were purchased from commercial vendors at the age of 6–8 weeks and transferred to standard housing conditions until desired age of 10, 12, 16 and 24 weeks were reached.

### Alteration of CALT

To study alteration of CALT under controlled pathological conditions, mice 12 weeks of age were stimulated following a modification of the previously published protocol [12]. Briefly, mice received repeated non-traumatic inoculation of 5 µl of a mixed solution of 0.25 µg/mL ovalbumin (OVA, Sigma Aldrich, Taufkirchen, Germany) or 0.25 µg/mL keyhole limpet hemocyanin (KLH, Calbiochem® Merck, Darmstadt Germany) together with 0.1 µg/mL choleratoxin B (CtB, Sigma Aldrich, Taufkirchen Germany) eight times over a period of two weeks. Control animals received no stimulation or 0.1 µg/mL choleratoxin B alone. At the end of stimulation the animals were euthanized and eyes were dissected and fixed as described above.

### Dependence on cervical lymph nodes and spleen

To investigate dependence of CALT on the presence of regional lymph nodes and the spleen, mice aged 10 weeks were anesthetized and either cervical lymph nodes of the right neck side or the spleen were removed. Cervical lymph nodes on the left remained untouched as internal controls. After 7 days of recovery animals were stimulated on both eyes with either OVA/CtB or KLH/CtB following the protocols described above. One day after the final stimulation, animals were euthanized and eyes were fresh-frozen in liquid nitrogen for definite histological assessment.

### Particle transport within CALT

To demonstrate particle transport, either fluorescing microspheres (200 µm, Fluoresbrite YG Microspheres, Polyscience Inc., Warrington PA, USA) or fluorescing heat-inactivated *E. coli* (BioParticles®, Molecular Probes,Leiden, Netherlands) were applied topically following antigen-challenge with KLH/CtB using the protocol described above. After 30 minutes and 60 minutes eyes were dissected as described and either fixed in a mixed solution of glutaraldehyde and formalin or fresh frozen in liquid nitrogen. Some animals that received microspheres were examined using intravital two-photon microscopy to detect fluorescing microspheres in different mucosal compartments *in vivo*.

### Intravital immune imaging

Prior to intravital two-photon microscopy, CALT was induced as described above. Imaging experiments were conducted 3 days after the last stimulation. Animals were anesthetized using intraperitoneal infusions of Fentanyl (Bayer Health Care, Leverkusen Germany), Midazolam (Curamed, Karlsruhe, Germany) and Medetomidine (Pfizer, Karlsruhe, Germany). To maintain sufficient blood oxygen levels mice received a tracheotomy and were ventilated automatically (MiniVent, FMI, Seeheim-Oberbeerbach, Germany). The animals were placed in a heated animal holder (MouseFix, Steven GmbH, Ochtrup, Germany) and placed below the two-photon microscope. Body temperature was maintained at 37°C by a heating mat attached to the animal holder. Blood oxygen levels and pulse rate were monitored throughout the experiment using a noninvasive infrared probe (MouseOx, Starr Life Science, Oakmont, PA, USA).

### Immunohistochemistry and electron microscopy

Fresh frozen tissue was serial sectioned using a Leica CM3050 Kryostat (Leica Microsystems, Wetzlar, Germany). Sections of 10–12 µm thickness were stained using a panel of antibodies (Tables 1 and Table 2) and counterstained with Hoechst-dye (Sigma, Taufkirchen, Germany). Images were recorded using a confocal microscope (Zeiss LSM 510 Meta, Jena, Germany). To detect particles within the tissue, thick sections (30 µm) were cut to record confocal image stacks. Thick sections were used to detect only particles within the tissue and to avoid misinterpretation of superficial particles that were dislocated by the sectioning process. By this, particles located at the cutting plain were excluded from analysis.

**Table 1:** Antibody staining panel.

Antibody-specificity	Clone	Source
Anti-CD 4	GK1.5	Santa Cruz Biotech, CA, USA
Anti-CD 8	53-6.72	Abnova, Taiwan
Anti-CD 45R/B220	RA3-6B2	BD Pharmingen, Germany
Anti-CD 25	PC61	BD Pharmingen, Germany
Anti-FoxP3	MF-14	BioLegend, CA, USA
Anti-CD 11c	N418	AcrisAntibodies, Germany
Anti-FDC	FDC-M1	BD Pharmingen, Germany
Anti-MHC II	ER-TR2	Novus Biologicals, UK
Anti-CD 31	MEC 13.3	BD Pharmingen, Germany
Anti-Lyve1	Polyclonal	AcrisAntibodies, Germany
Anti-ICAM-1	BE29G1	Leinco Technologies Inc., USA
Anti-ICAM-2	3C4 (mIC2/4)	LifeSpan Biosciences, USA
Anti-MadCAM 1	MECA-367	Antibodies-online GmbH, Germany
Anti-VCAM 1	Polyclonal	Santa Cruz Biotech Inc., USA

**Table 2:** Antibody combinations used and related cellular targets (note that antibodies do not stain the quoted cells exclusively).

Primary Antibodies	Target
CD 4	CD4+ T-helper cells
CD 8	CD8+ Cytotoxic T-cells
CD 45R/B220	B-cells
CD4/CD8/CD 25/FoxP3	Regulatory T-cells
CD 11c/MHCII	Dendritic cells
FDC	Follicular Dendritic cells
CD 31	Blood vessels/Endothelial cells
Lyve1	Lymphatic endothelial cells
ICAM-1	Endothelial cells, lymphocytes, epithelial cells, Dendritic cells, keratinocytes
ICAM-2	Lymphocytes, platelets, monocytes
MadCAM 1	Directs leukocyte migration to mucosal tissues
VCAM 1	Upregulated on inflamed/activated endothelial cells

For transmission electron microscopy tissue was fixed with a solution of paraformaldehyde and glutaraldehyde and contrasted with osmium-tetroxide. Tissue was embedded in resin (Araldite, Merck, Darmstadt, Germany). Serial sections were prepared using an UltraCut E ultramicrotome (Reichert Jung, Wetzlar, Germany) and contrasted with uranyl acetate and lead citrate.

Examination was conducted using a JEOL electron microscope (JEM, Eching, Germany).

### Semiautomatic quantification of cells/follicle/slide

For each time point 2–3 immunohistochemical stained lymphoid follicles were analyzed using the cell counter plugin of free available histomorphic analysis software (Image J). Overall cell number of follicles (stained with Hoechst-dye) was counted as well as cells stained with the antibodies against CD4, CD8, B220 or FDC. Furthermore cell ratios of specific cell types related to overall cell number of the follicle were calculated. Mean +/− standard deviation were calculated from 2–3 ratios. Finally overall cell number of all analyzed slices of each time point were added and divided through the number of slices for average number of cells per follicle/slide per time point.

### Two-photon microscopy

The two-photon microscope (DermaInspect®, Jenlab, Jena, Germany) used in this study was equipped with a tunable infrared femtosecond laser (Wide Band MaiTai, Spectra Physics, USA, 720–920 nm tuning range). The DermaInspect contained a computer-controlled beam attenuator, a shutter, and a two axis galvoscanner. A 20× objective with 1.4 mm working distance (W Plan-Apochromat 20×, 1.0 DIC Vis-IR, Zeiss, Göttingen, Germany), which was focused by a piezodriven holder, was used. Larger scale motions of the sample in x-, y- and z-directions were performed by computer-controlled stepper-motors (Owis GmbH, Staufen, Germany). The autofluorescence was detected by a standard photomultiplier module (H7732, Hamamatsu, Herrsching, Germany) after passing through a beam splitter (Chroma 640 DCSPXR, AHF analysentechnik AG, Tübingen, Germany) and a short-pass filter (BG39, Schott, Mainz, Germany), resulting in a detection bandwidth from 380 nm to 530 nm (FWHM), which could not detect the SHG signal for an excitation shorter than 760 nm.

The animals were anesthetized and positioned in the fixation stage as described above. Each animal was oriented with the eye facing upright and the working distance of the objective was bridged with Vidisic-Gel (Bausch u. Lomb, Berlin, Germany), which simultaneously prevented desiccation of the ocular surface.

Autofluorescence 2D time series (x,y,t) of CALT regions were recorded at 710–730 nm excitation wavelength over periods of up to 90 minutes for each time series.

### Tracking of lymphocytes

Cells were manually tracked using Imaris 7.2 software including a tracking module (Bitplane, Zurich, Switzerland, Video S3) as published previously by our group [15]. Individual autofluorescent cells were identified and marked, followed by applying the tracking function. By this individual tracks were generated by the tracking module of Imaris and exported to Excel.

### Statistical analysis

Statistical analysis included univariate ANOVA (analysis of variance) with Post Hoc test LSD for the general expression of CALT and the number of CALT per eye. Gaussian distribution of data was analyzed using Kolmogorov-Smirnov test. Statistical analysis of lymphocyte tracking included ANOVA (One-way analysis of variance) and Bonferroni Multiple Comparison Test. (p-values were considered to be significant below p<0.05). Statistical analysis were conducted using SPSS (SPSS Inc., IBM, Germany, version 21).

## Results

### Development of CALT follows a coordinated time course

To study the development of CALT under conditions without experimental antigen challenge, mice were kept under specific pathogen free animal housing conditions. Based on distinguished studies of Isaacson and co-workers on MALT, in particular MALT lymphomas [16]–[18], we defined necessary components of an organized lymphoid aggregate to be accounted as conjunctiva-associated lymphoid tissue in the model used. As absolute requirements we defined: i. lymphoepithelium that lacks goblet cells, ii. lymphoid follicle with a T-zone containing CD4+ and a B-zone with B-cells and FDCs, iii. Adjacent high-endothelial venules (HEVs) and lymphatics. According to this definition, analysis of the number of CALT follicles in the nictitating membranes was conducted. Although it is likely that diffuse lymphoid tissue of the conjunctiva has a potential immunological role, this study was solely focused on organized CALT because of its unique spatial organization [1].

**Figure 1:**
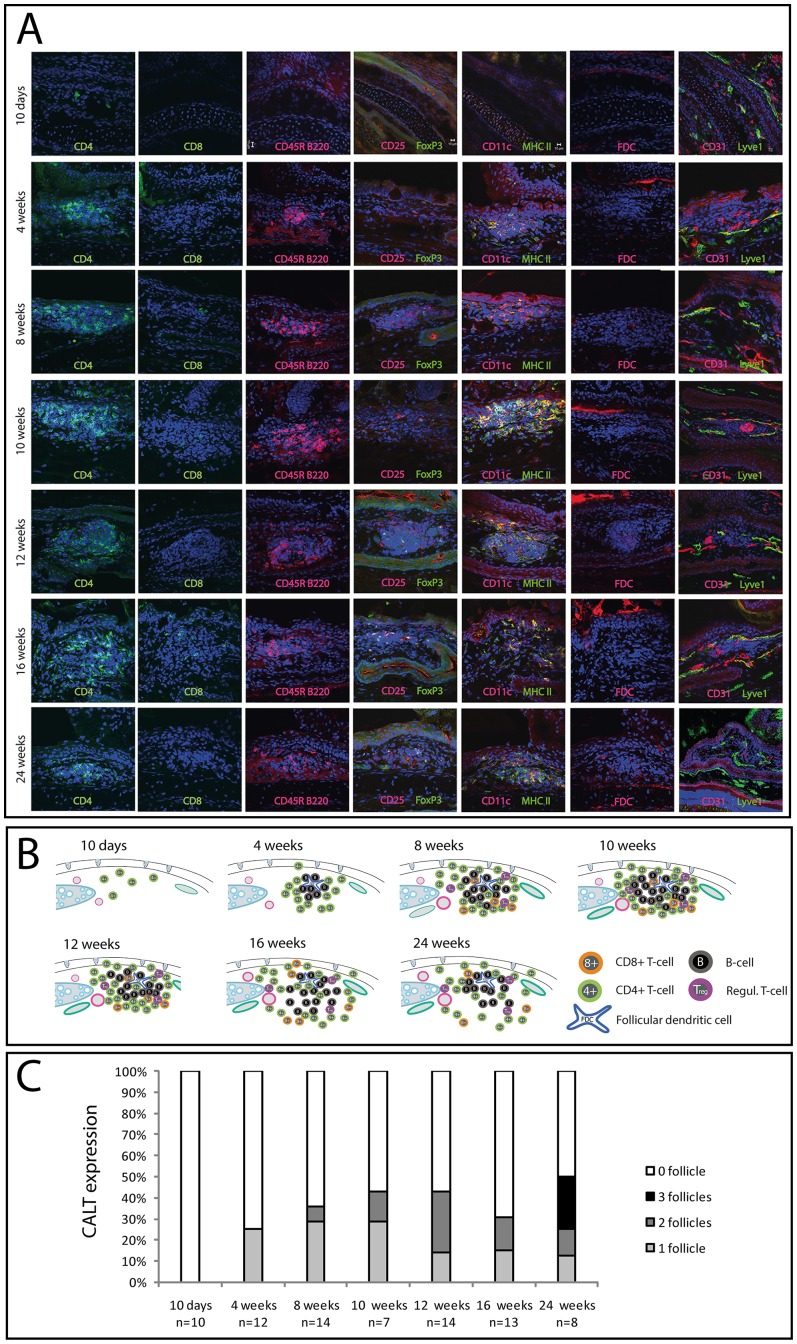
Development of CALT under SPF housing conditions. A) Immunhistological analysis of conjunctiva-associated lymphoid tissue (CALT). Mice aged 10 days until 24 weeks, kept under SPF housing conditions, were investigated using a panel of antibodies as described in table 1. Spatial distribution of lymphocytes, dendritic cells and follicular dendritic cells was analyzed. B) Schematic CALT development: 10 days after birth only CD4+ T-cells were sparsely present, followed by an influx of B-cells and formation of first follicles at 4 weeks of age. At 8 to 12 weeks CD8+ T-cells and CD4+CD25+ Tregs appeared, altogether forming a complex lymphoid follicle. After 16 weeks of age spatial organization diminished. C) CALT expression rate: CALT was not present at 10 days of age. 25% of the eyes at 4 weeks of age contained CALT, with a further increase to 43% CALT at 10 and 12 weeks of age. This was followed by a decrease to 31% at 16 weeks and an increased to 50% at 24 weeks of age. Numbers of follicles increased until 25% of the eyes contained 2 follicles at 12 weeks of age. At 24 weeks of age 25% of the eyes contained 3 follicles (n = number of eyes).

At 10 days postnatal mice that had their eyelids still closed, demonstrated sparsely CD4+ T-cells present in the nictitating membrane with few lymphatics and blood vessels. At 4 weeks postnatal, CD4+ T-cells formed a diffuse infiltrate containing few B-cells and CD4+ CD25+ regulatory T-cells (Tregs). In 25% of the eyes organized lymphoid follicles were observed and increasing numbers of lymphatics and blood vessels were noted. At 8 weeks of age organized lymphoid follicles were detected in 35% of the eyes that demonstrated a lymphoepithelium, a T-zone with CD4+ T-cells, CD8+ T-cells and CD4+CD25+FoxP3+/− Tregs and a central B-Zone with few FDCs. Large blood vessels and a dense network of lymphatics were located in close proximity to the follicle. At 12 weeks of age CALT expression increased to 43%, although T- and B-zones lost their strict spatial organization (Figure 1A+B).

The number of follicles/eye also increased during aging (Figure 1C). At 4 weeks the eyes contained only 1 follicle in up to 25% of the eyes whereas at 10 weeks of age the number of follicles increased to 1 follicle/eye in up to 29% and two follicles/eye in up to 14% of the eyes. At 12 weeks of age two follicles/eye were present in 29%. At 16 weeks of age overall CALT expression and numbers of follicles decreased, followed by a markedly increase to 50% at the age of 24 weeks. Here >20% of the eyes contained 3 follicles (Figure 1C).

### Migration receptors are expressed at all time points during CALT development

As CALT development depends on specific cellular migration into the target tissue, a process strongly depending on the presence of migration receptors, we investigated the expression of migration receptors VCAM-1, MadCAM-1, ICAM-1 and ICAM-2 that were described to be crucial for development of organized MALT [19] by immunohistochemistry.

**Figure 2:**
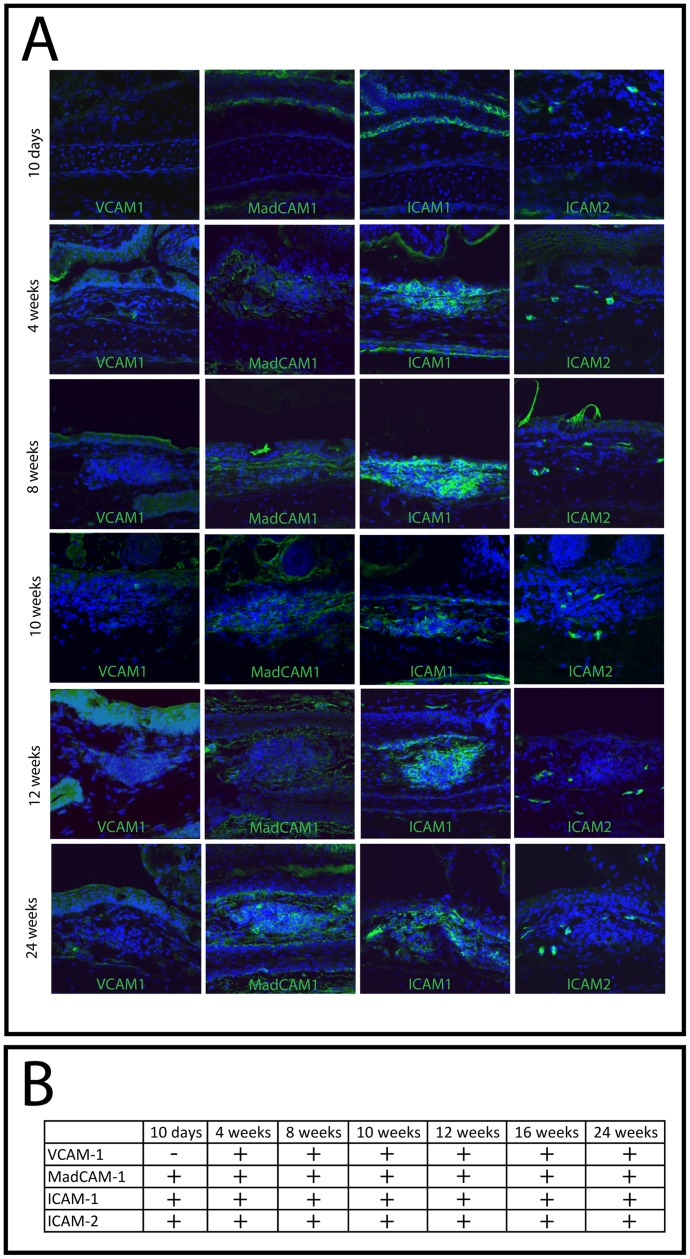
Expression of migration receptors. A) Immunohistochemistry of migration receptors at different time points during development. At 10 days of age no CALT was present, therefore representative sections from the nictitating membrane were chosen. For all other time points present in the epithelium, subepithelial space and within lymphoid follicles. B) Time dependent analysis of migration receptors demonstrated presence of all receptors at all time points, except that VCAM-1 was not present at 10 days of age.

This analysis demonstrated a presence of all adhesion molecules tested at all time points except that VCAM-1 was not detectable at 10 days of age (Figure 2). The staining pattern revealed antibody binding within the epithelium and submucosa. CALT follicles were positive for all migration receptors from week 4 when first follicles were present.

### Role of animal housing conditions

**Figure 3:**
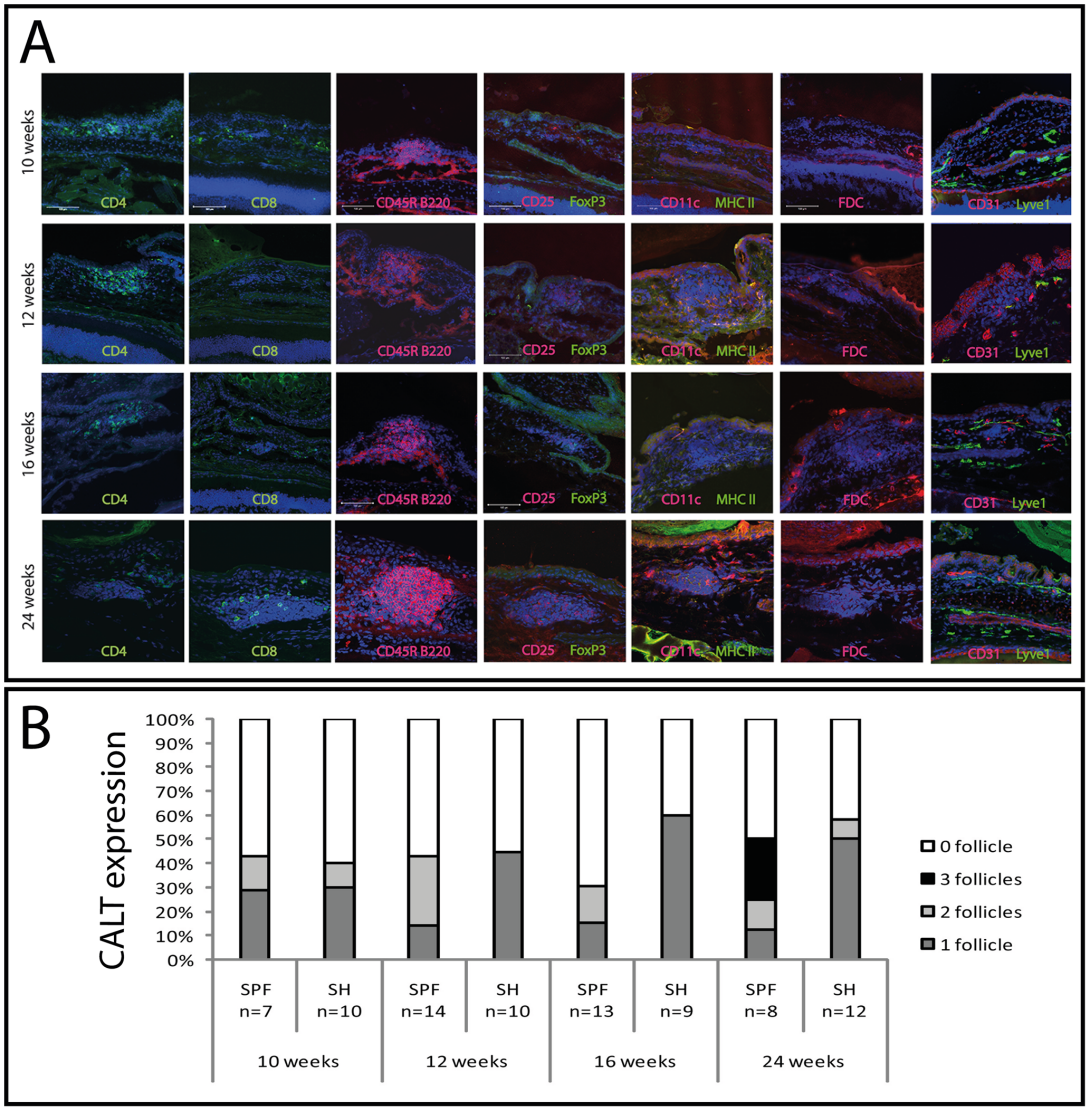
CALT under standard housing conditions. Immunhistological staining of CALT at 10–24 weeks that were kept under standard housing conditions were investigated using a panel of antibodies as described in table 1. Spatial distribution of lymphocytes, dendritic cells and follicular dendritic cells was analyzed. A) Comparison of CALT expression in mice kept under standard housing conditions with concordant time points of animals kept under SPF housing conditions (expression rates from figure 1). Statistically CALT expression was similar in both housing conditions. (SPF = specific pathogen free; SH = Standard housing conditions; n = number of eyes examined. Differences were statistically not significant, p>0.05).

To investigate the potential role of animal housing conditions with a presumed higher antigen presence in standard animal housing than under SPF conditions, mice were kept under standard housing conditions for at least 2 weeks and then analyzed for the presence of CALT (Figure 3A). At the concordant time points 10, 12, 16 and 24 weeks of age animals from standard housing conditions did not have significant higher expression of CALT or more follicles in the nictitating membrane of the eye, although at 16 and 24 weeks of age a trend towards more CALT under standard housing conditions is visible (Figure 3B). A first approach to quantify overall cellular numbers in CALT from both housing conditions demonstrated mean cell numbers between 128 (±42) and 240 (±78) cells/follicle/histological section under standard housing conditions (Figure S1A) and mean cell numbers between 226 (±69) and 281 (±88) cells/follicle/histological section under SPF housing conditions (Figure S1B). Interestingly under standard housing conditions at 16 weeks of age the amount of B-cells increased to almost 70%, whereas CD4+ T-cell ratios varied around 30–45% under both housing conditions. Overall CD4+ T-cells were more dominant as CD8+ T-cells.

In summary, CALT shows an orchestrated development with migration receptors present at all time points investigated. Animal housing conditions had no influence on the expression of CALT.

### Topical antigen-challenge enhances CALT expression

**Figure 4:**
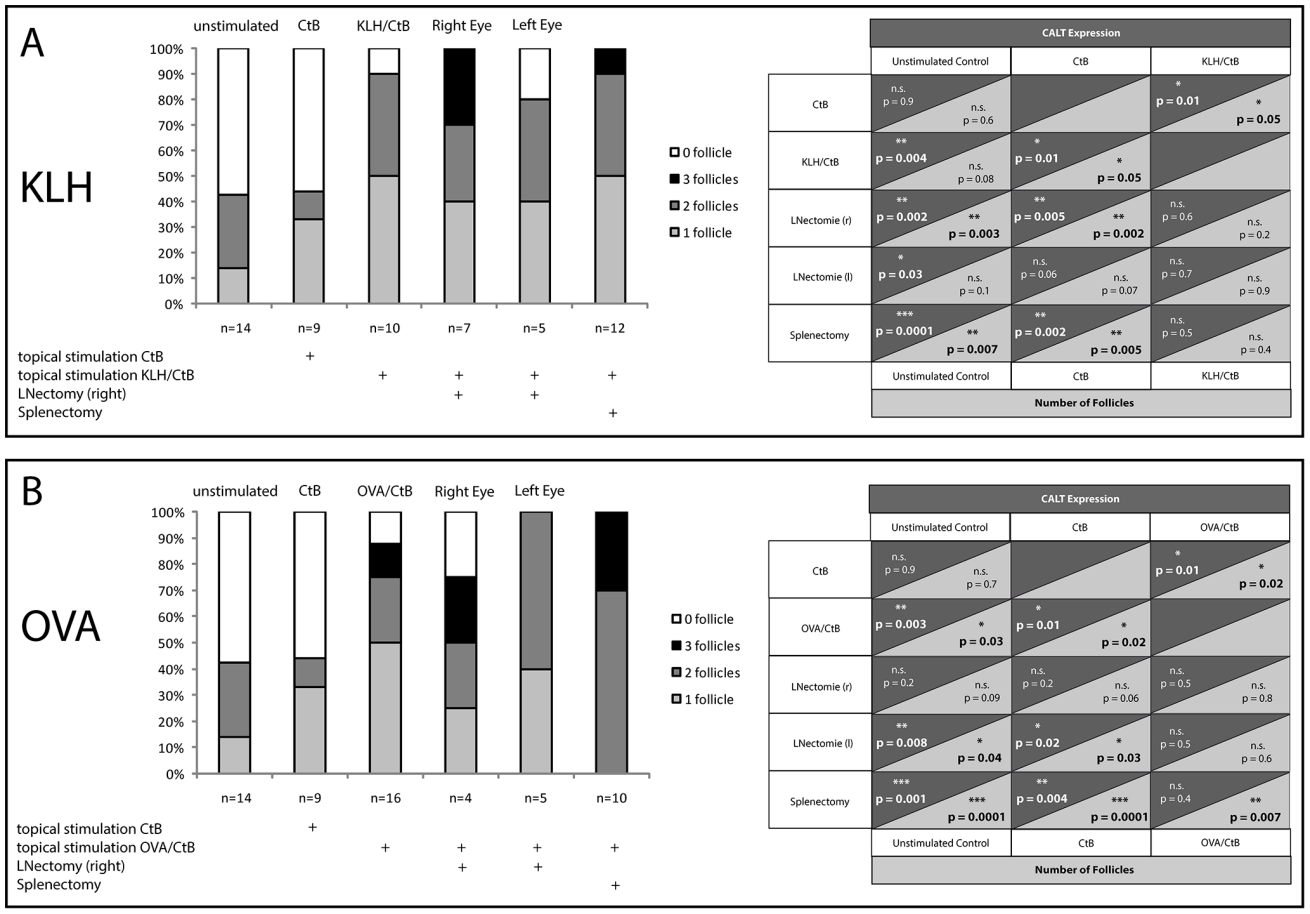
CALT expression at 12 weeks of age, following repeated antigen-challenge with keyhole limpet hemocyanine/choleratoxin B (KLH/CtB) or ovalbumin/choleratoxin B (OVA/CtB) after splenectomy or lymphadenectomy. A) Topical stimulation with KLH/CtB resulted in significant increase of CALT (90%) in comparison to unstimulated control or control with choleratoxin B alone. Cervical lymphadenectomy of the right side followed by topical stimulation in both eyes lead to 100% CALT expression in right eyes. Left eyes contained follicles in 80%. After splenectomy topical KLH/CtB stimulation resulted in CALT expression of 100%. There was no statistical difference between KLH/CtB and animals that received lymphadenectomy or splenectomy. Experiments were performed twice. B) Topical stimulation with OVA/CtB resulted in a significant increase of CALT expression to 88% of eyes with follicles in comparison to controls. Cervical lymphadenectomy of the right side followed by topical stimulation in both eyes lead to 76% CALT expression in the right eye. Left eyes contained follicles in 100%. After splenectomy topical KLH/CtB stimulation resulted in CALT expression of 100%. There was no statistical difference in CALT expression or number of follicles between OVA/CtB and animals receiving lymphadenectomy or splenectomy. Experiments were performed twice. Statistical analysis (table 1 and table 2) was performed using univariate ANOVA (with LSD Post Hoc test) between all groups for CALT expression and number of follicles per eye. Significant values were compared to unstimulated control, CtB stimulated control and OVA/CtB or KLH/CtB stimulated group. (p>0.05 n.s., p≤0.05 *, p≤0.01 **, p≤0.001 ***).

As animals from standard-housing conditions demonstrated no statistical difference in the expression rate of CALT in the eyes in comparison to animals from SPF-conditions, we hypothesized that an exceeding amount of antigen that challenges the eye topically may alter CALT expression. We therefore applied topical antigens (KLH+choleratoxin B (CtB) or OVA+cholera toxin B (CtB)) onto the conjunctiva (Figure 4). In comparison to unstimulated controls from SPF-facilities and CtB stimulated controls, the increase of CALT was statistically significant. An overall expression rate of 90% CALT in KLH/CtB and 88% CALT in OVA/CtB stimulated animals aged 12 weeks was obtained. In KLH/CtB stimulated animals 50% had one follicle/eye and 40% had 2 follicles/eye. In OVA/CtB stimulated mice 50% of the animals had 1 follicle/eye, 25% had 2 follicles and 13% contained 3 follicles per eye.

### Cervical lymphadenectomy and splenectomy enhance CALT expression

Regional lymph nodes play a crucial role in initiating a secretory immune response in a number of ways such as locally expanding effector cell populations following antigen-specific activation of immune cells within mucosal surfaces [20]. As topical antigen-challenge significantly increased the expression of CALT, we tested the hypothesis, that this increase would be further enhanced once ipsilateral regional lymph nodes or the spleen were removed prior to topical antigen challenge.

One group of animals received unilateral lymphadenectomy only on the right side of the neck, whereas both eyes were equally challenged with KLH/CtB or OVA/CtB. A second group of animals received a splenectomy prior to antigen challenge in both eyes.

Cervical lymphadenectomy of the right side followed by topical KLH/CtB stimulation increased the total number of CALT/eye to 100% in the right eye as well as an increase in overall number of follicles, although this increase was not statistically different to KLH/CtB stimulated animals. Left eyes demonstrated 80% CALT. Splenectomized mice that received KLH/CtB showed 100% CALT and an increase in overall numbers of follicles. 50% of the animals had 1 follicle/eye, 40% had 2 follicles and 10% had 3 follicles per eye (Figure 4A+B).

Lymphadenectomy of the right cervical side followed by topical stimulation with OVA/CtB led to 76% CALT expression in the right eye and a non-significant increase in overall number of follicles in comparison to controls. Left eyes of these animals however exhibited 100% follicles. Splenectomy prior to topical OVA/CtB stimulation increased non-significantly overall expression of CALT to 100%, whereas the number of follicles was significantly increased in comparison to CtB stimulated and non-stimulated controls (Figure 4C+D).

### Topical antigen-challenge leads to T-cell activation in CALT

**Figure 5:**
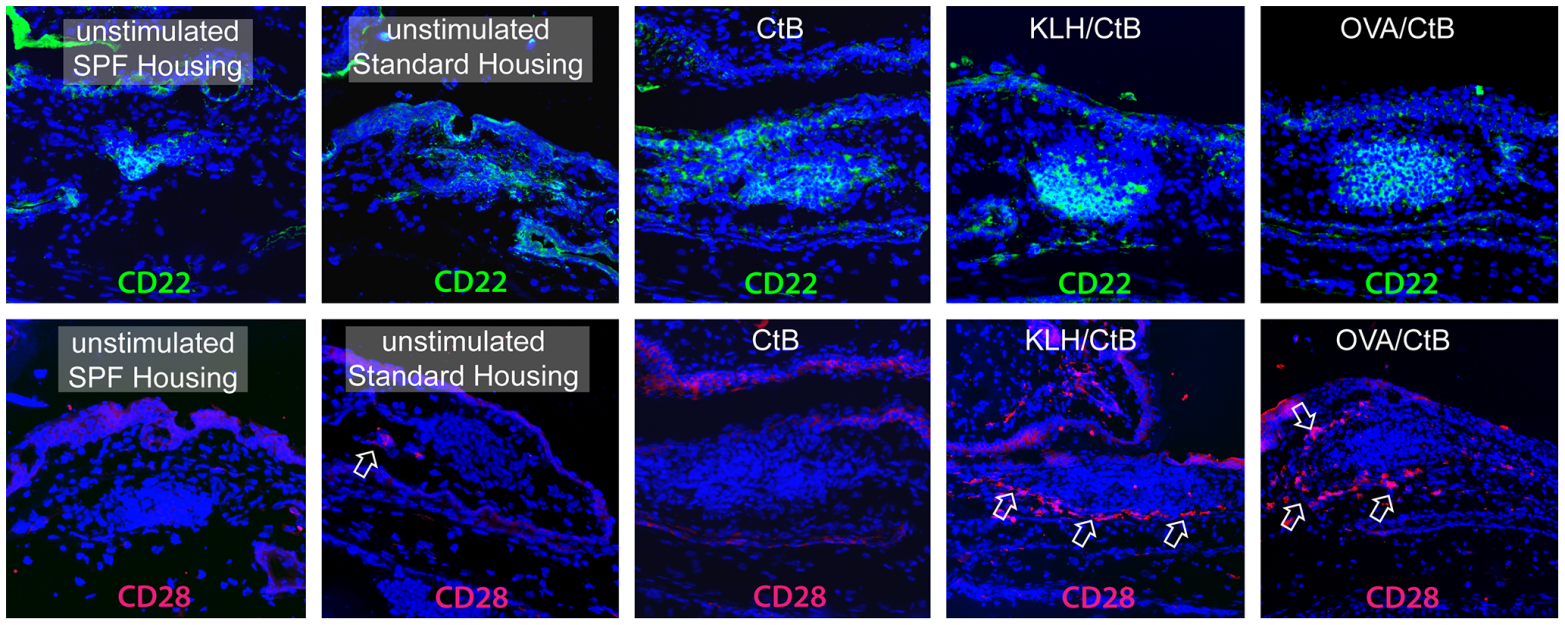
B-cell and T-cell activation markers in CALT following stimulation with KLH/CtB and OVA/CtB in 12 week old animals. CD22 positive B-cells were found in all sections from all animals. CD28 positive T-cells were restricted to animals stimulated with KLH/CtB or OVA/CtB respectively.

Immunohistochemical staining with CD22 as an B-cell activation marker [21] and CD 28 as a T-cell activation marker [22] demonstrated activated B-cells in control animals from both housing conditions that received no antigen-challenge or CtB alone and animals that were challenged with KLH/CtB or OVA/CtB. Activated T-cells were only detectable in animals that were challenged with KLH/CtB or OVA/CtB (Figure 5).

### Particle-uptake within CALT

**Figure 6:**
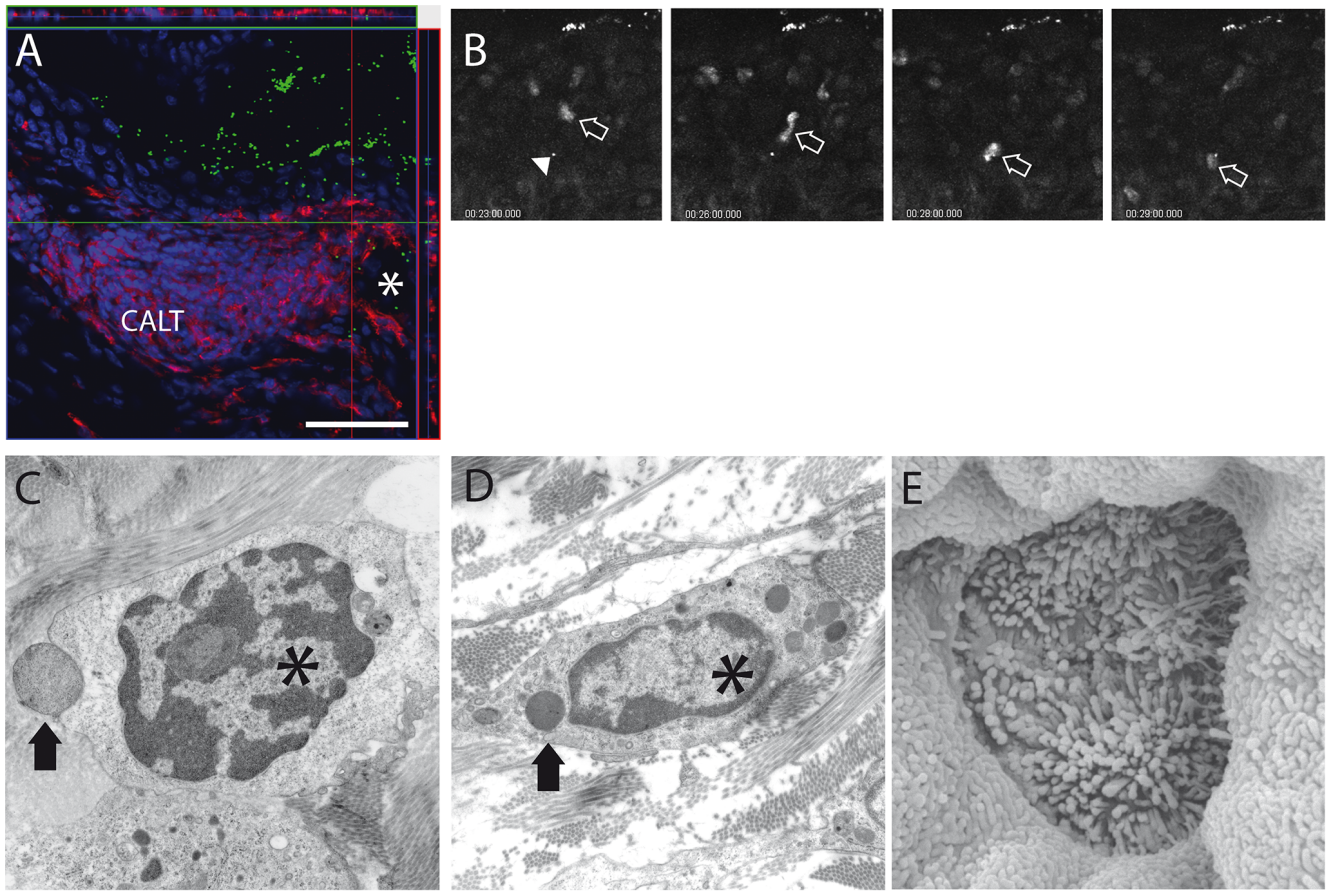
Particle-uptake within CALT. A) Fluorescing *E. coli* bacteria (green) were transported through the lymphoepithelium of CALT and detected close to MHC II positive cells (red) within CALT and around and within lymphatic vessels (asterisk) 60 minutes after application. (*Ex vivo* confocal microscopy, image stack, steps 1 µm, scale bar 55 µm). B) Intravital two-photon microscopy of CALT lymphoepithelium. An intraepithelial fluorescent microsphere (arrowhead) is approached by a DAPI-labeled lymphocyte (arrow). (Time course in minutes). C+D) Transmission electron microscopy of *E. coli* particle (arrow) during approach (C) and within (D) a macrophage (asterisk). E) Scanning electron microscopy of lymphoepithelium. Cellular surface villi with typical features of M-cells.

One of the unique features of mucosa-associated lymphoid tissue is the ability to sample particular (e.g. bacteria) or non-particular (e.g. soluble) antigens from the mucosal surface and transport these across the epithelium by specialized M-cells [23]. In concordance to other animal models [8], [24], [25], in the mouse, fluorescent microspheres as well as heat-inactivated *E. coli* were translocated preferentially via the lymphoepithelium of CALT. Both particles and bacteria were localized by means of confocal laser-scanning (Figure 6A) and two-photon microscopy (Figure 6B, Video S1) within the epithelium and subepithelial space and in close proximity to the follicle. Particles and bacteria were both detected 60 minutes after initial topical application onto the surface, demonstrating a rapid uptake. Electron microscopy of translocated *E. coli* documented phagocytosis of bacteria by macrophages within the subepithelial space (Figure 6C+D). Intravital two-photon microscopy using fluorescent microspheres demonstrated particles laying within the wall of lymphatic vessels as well as sporadic cell-particle interactions without signs of cellular arrest (Figure 6B, Videos S1+S2).

Ultrastructural analysis of CALT demonstrated features of M-cells within the epithelium, as demonstrated by Seo et al. [26]. This included cells with typical surface pattern (Figure 6E) and basolateral pockets (Video S2). However, in contrast to other animal models, M-cells in the nictitating membrane of the mouse seem to be reduced in number and different in shape, both features that are currently under further examination.

### CALT demonstrates pronounced cellular dynamics in different compartments

**Figure 7:**
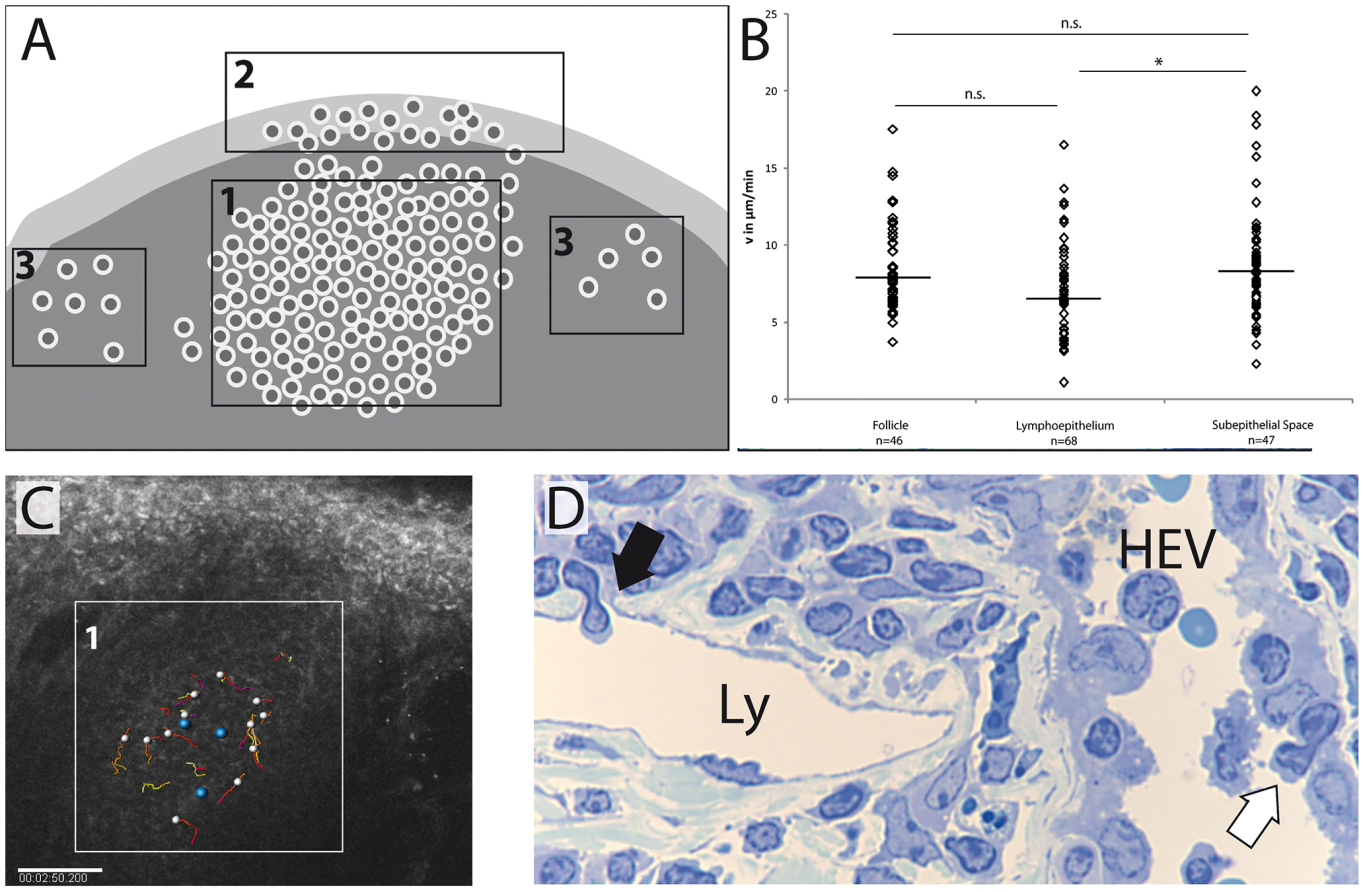
Classification of CALT compartments and according cellular velocities. A) Schematic drawing of CALT microcompartment with CALT follicle (1), lymphoepithelium (2) and scattered lymphocytes within subepithelial space (3). B) Cells within the lymphoid follicle featured median velocities of 8.0 µm/min (n = 46 cells, 874 individual measurements). Intraepithelial lymphocytes (lymphoepithelium) demonstrated median velocities of 6.7 µm/min (n = 68 cells, 2101 individual measurements) whereas cells within the subepithelial space demonstrated median velocities of 8.4 µm/min (n = 47 cells, 2416 individual measurements). Statistical analysis demonstrated significant differences of the velocities between lymphoepithelium and subepithelial space (*p<0.05). The data derives from 6 individual experiments with 9 time series/separate sets of data. Statistical analysis included One-Way ANOVA (p = 0.02) for testing of normal distribution, followed by Bonferroni Multiple Comparison Test. Significance values below p<0.05 were considered to be significant. Bartlett-Test was used to analyze data variances (p = 0.4). (p>0.05 n.s., p≤0.05 *). C) Intravital two-photon microscopy of CALT and consecutive tracing of individual cells within the follicle (zone 1, compare to 7A). White spheres represent motile cells, blue spheres represent non motile cells with distinct differing autofluorescence properties and dendritic cell-like morphology (compare supplemental Video 3). D) Lymphoid vessel (Ly) and high endothelial venule (HEV) located in close proximity to a CALT follicle demonstrate cellular transmigration. A lymphocyte migrates into a lymphatic vessel (black arrow), whereas another lymphocyte migrates from a high endothelial venule (white arrow).

Besides the activation of specialized immune cells, lymphoid tissue function largely depends on the migratory ability of these cells to be able to contact other immune cells and to reach desired tissue compartments including germinal centers, blood- or lymphatic vessels, and mucosal lamina propria. Using intravital two-photon microscopy based on tissue autofluorescence we were able to analyze for the first time real-time dynamics of immune cells within CALT [27]. In this study we differentiated three compartments within CALT, namely the entire lymphoid follicle, lymphoepithelium and adjacent subepithelial space (Figure 7A) to analyze cellular velocities in the absence of tissue inflammation. Intravital two-photon microscopy revealed rapid cellular dynamics in all three compartments investigated (Videos S1+S3). In accordance to immunohistochemical analysis and previously described intravital morphometric features such as diameter and shape [12], the majority of mobile cells within lymphoepithelium, follicle and subepithelial space were lymphocytes, namely B- and T-cells (Figures 1+3). Cellular velocities were 8.0 (±2.9) µm/min within the follicle and 8.4 (±3.6) µm/min in the subepithelial space. Intraepithelial cells were significantly slower than subepithelial cells demonstrating 6.7 (±3.3) µm/min (Figure 7B+C). Cellular exchange in between subepithelial space and follicle as well as follicle and epithelium was observed frequently (Video S2). Interestingly cells within basolateral pockets demonstrated rapid rotational movement (Video S2). Transmigration of cells from blood vessels or into lymphatic vessels was not recorded, but depicted in conventional histological analysis (Figure 7D).

## Discussion

The ocular surface is constantly challenged by potential pathogens. To maintain its integrity defense mechanisms such as lymphoid tissue are present in the mucosa as diffuse and organized conjunctiva-associated lymphoid tissue (CALT). Due to the unique spatial organization of organized lymphoid tissue (CALT) much has been hypothesized on the function of CALT. In fact, only little is known on CALT function, mainly due to technical restrictions within the animal models used so far. Following the introduction of a mouse model, this study was set up to investigate the function of CALT including aspects of CALT development, dependence on antigen-challenge and immune cell migration.

The data presented demonstrate that development of CALT in the mouse follows a defined time course, similar to humans. The finding that follicles are lacking at 10 days after birth, when eyelids are still closed, together with a significant increase of CALT following experimental antigen-challenge demonstrates an antigen-dependent inducibility of CALT. The presence of homing receptors MadCAM-1, ICAM-1 and ICAM-2 as well as lymphatics and blood vessels at 10 days of age and closed eyelids without the presence of foreign antigen, however implicates early structural predisposition of the conjunctiva for immigration of immune cells.

The finding of increased numbers of follicles in animals kept under SPF-conditions aged 24 weeks was unexpected, as in healthy human adults CALT follicles decrease after a physiological peak at adolescence. Obvious reasons for the detected increase such as ocular infection were excluded as demonstrated by normal health reports and normal morphology of the eyes. In this context it has been described that during aging the overall number of lymphoid follicles in the intestine of mice increases [28] and this so called *immunosenescence* may contribute to autoimmunity and inflammation, a process termed *inflamm-aging* [29]. Considering CALT, it is of particular interest whether increasing numbers of follicles in aged mice may also resemble a form of *immunosenescence* and *inflamm-aging*. Further studies using aged animals will address this topic and may open up a new field in the understanding of ocular surface immunology in relation to age. As no statistical difference between housing conditions were seen, no consequence in terms of choosing control animals from either housing condition is deduced for further studies.

In comparison to other mucosa-associated lymphoid tissues the lack of lymphoid follicles at birth is not a common finding. For example, Peyer's patches (PPs) in the intestine are already present prenatally and fully develop within the first week after birth [30], [31]. Bronchus-associated lymphoid tissue (BALT) instead is absent in healthy mice and humans in general but it is inducible following infection or inflammation [32]. Tear duct-associated lymphoid tissue (TALT) develops parallel to CALT but, in contrast, contains B-cells at very early stages of development along with lymphatic inducer cells (LTi-cells) which have been shown to generate the necessary environment for the development of organized lymphoid tissues [33].

For the first time this study demonstrates the presence of CD4+CD25+FOXP3+/− regulatory T-cells (Tregs) in conjunctival follicles (Figure 1A+3A). This finding strongly supports the hypothesis that CALT has immunoregulatory properties. In this context it is likely that the clinical described increase of CALT in autoimmune diseases such as dry-eye is not only related to an increased immune response towards currently unknown self-antigen, but also related to an increasing need of immune regulation by Tregs [34].

Besides immunoregulation a presumed main function of CALT is to transport antigenic matter across the epithelial barrier and to initiate immune responses in conjunction with regional lymph nodes. Like other species such as rabbits, turkeys and chicken, CALT lymphoepithelium in mice transports particles and bacteria from the ocular surface preferentially into the subepithelial space (Figure 6). Immunologically inert particles and bacteria are approached by immune cells (Figure 6B+C) and bacteria are phagocytosed by macrophages (Figure 6D). The further steps of antigen-processing, presentation and migration of CALT-derived immune cells to local lymph nodes remain to be investigated. In this context, it was uncertain, whether regional lymph nodes or the spleen were functionally connected to CALT. Experiments using the protozoal parasite *T. cruzi* as a topical conjunctival stimulant revealed that the parasites were only present within the unilateral lymph node [35] and experiments tracing OVA applied topically to the conjunctiva were not able to detect antigen within the spleen [36]. Performing unilateral lymphadenectomy or splenectomy led to a strong increase in numbers of follicles in the nictitating membrane of the eye following topical antigen challenge (Figure 4), however the role of the lacking spleen in this context is unclear and currently under investigation. As in OVA/CtB-stimulated animals, CALT also increased in the eye, which was related to the untouched side of the neck, suggesting a cross-drainage of antigen. Based on these results we hypothesize, that an increase of CALT following lymphadenectomy or splenectomy may therefore be an increase in local immune-competency in order to better protect the ocular surface. Both development and alteration of CALT rely on migration of immune cells from and to the conjunctiva. Recently Liang et al. investigated rabbits by means of confocal laser scanning microscopy (CLSM) in order to analyze dynamic processes that occur during inflammation of the ocular surface and its related changes of CALT [37]. As CLSM fails to monitor single cell migration and according velocities inside the tissue in real time, we used intravital two-photon microscopy based on intrinsic tissue autofluorescence with the optional use of fluorescent dyes [3], [12], [27]. This approach enables real time investigation of dynamic immunological processes as demonstrated in lymph nodes and spleen in living animals [38], [39]. Cellular velocities did not differ significantly within the follicle and the subepithelial space (8.0 vs. 8.4 µm/min) in contrast to intraepithelial lymphocytes that migrated at significantly slower velocities (6.7 µm/min) most likely due to higher epithelial tissue resistance. Non-migrating cells within the follicle featuring large cellular bodies with dendritic protrusions and strong autofluorescence signal (Video S1) were classified follicular dendritic cells according to the autofluorescence signal and the immunohistochemical *ex vivo* staining using an FDC antibody (Figures 1A+2A). Velocities measured in our experiments were in the range of velocities of T-cells (10.8 µm/min) or B-cells (6.4 µm/min) in lymph nodes [40]. As we used either intrinsic tissue autofluorescence or non specific fluorescent staining we were not able to differentiate B-cells from T-cells. However, histological analysis following intravital microscopy revealed a majority of T-cells in the subepithelial space and lymphoepithelium; therefore, velocities measured in these compartments are assigned to T-cells, and measurements within the follicle are assigned to mixed B- and T-cell subsets. Currently, experiments are underway to compare physiological migration pattern with velocities during inflammatory states such as dry-eye or acute ocular allergy.

In summary, the findings presented in this study demonstrate for the first time functional properties of CALT in terms of development, inducibility and adaption to antigen presence, connection to secondary lymphoid tissues and short term cellular dynamics. The acknowledgment of CALT as an immunological interface amends the understanding of immune processes taking place within the conjunctiva and closes the gap between well investigated fields of corneal immunity, conjunctivitis and function of regional lymph nodes, hereby forming a more precise concept of the ocular surface immune system.

## Supporting Information

Figure S1 **Cell counts during aging under standard housing and SPF housing based on immunohistochemical staining.** A) Total mean cells per follicle/slide under standard housing ranged from 128–240 (Standard housing). B-cells and FDCs/total cells per follicle/slide peaked at 16 weeks of age. B) Total mean cells per follicle/slide under SPF housing ranged from 226–281. B-cells/total cells per follicle/slide peak at 24 weeks of age, whereas CD4+ and CD8+ T-cells and FDCs remain stable at all time points.
(TIF)

Video S1 **Intravital two-photon microscopy of CALT.** Based on tissue autofluorescence a large lymphoid follicle is visible below a bright fluorescing epithelium. On both sides of the follicle large diameter vessels are visible. These vessels are presumed lymphatics as no red blood cells are located within the lumen and previously topically applied microspheres (bright white dots) are located within the vessel wall. Over a period of appr. 5.5 minutes rapid cellular migration is visible within the follicle, whereas three bright fluorescing cells with dendritiform shape do not demonstrate any movement (arrows FDC's).
(MOV)

Video S2 **Intravital two-photon microscopy of a basolateral pocket within the lymphoepithelium of CALT.** Based on tissue autofluorescence an intrapepithelilal pocket is visible containing several motile cells that demonstrate rotational movements. Below the pocket other cells migrate into and from the pocket (cellular traffic). In addition topically applied fluorescent microspheres (bright white dots) are located onto and within the epithelium.
(MOV)

Video S3 **Intravital two-photon microscopy of CALT (image series from video 1).** Based on tissue autofluorescence a large lymphoid follicle is visible below a bright fluorescing epithelium. Using Imaris Software including a tracking module, individual motile cells were manually marked and semiautomatically traced. Motile cells (red dots) are present as well as non-motile cells (blue dots) that correspond to bright fluorescing dendritiform cells.
(MOV)
