# Supplementary figures and images for: Development, Alteration and Real Time Dynamics of Conjunctiva-Associated Lymphoid Tissue
Source: PLoS One. 2013 Dec 20;8(12):e82355. doi: 10.1371/journal.pone.0082355 (PMC3869694; doi:10.1371/journal.pone.0082355)

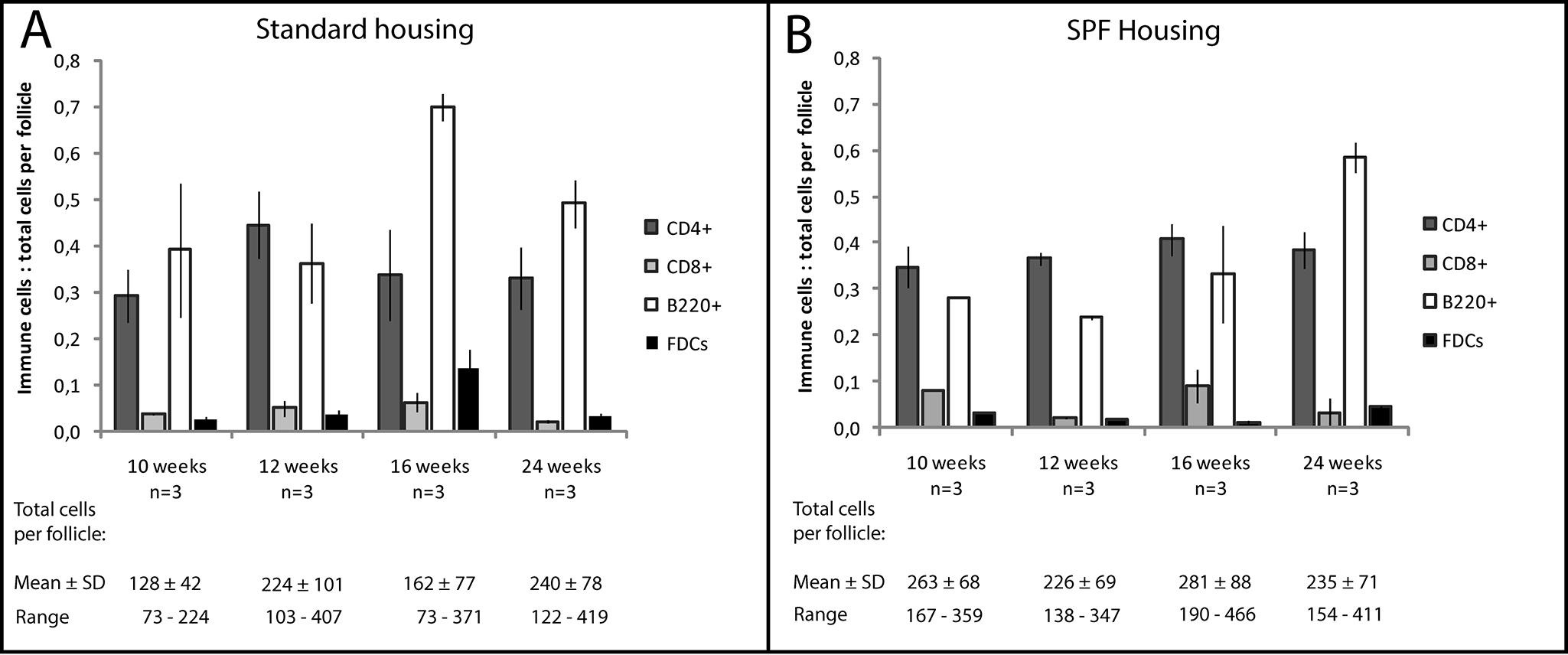

Supplement: Figure S1 — Cell counts during aging under standard housing and SPF housing based on immunohistochemical staining. A) Total mean cells per follicle/slide under standard housing ranged from 128–240 (Standard housing). B-cells and FDCs/total cells per follicle/slide peaked at 16 weeks of age. B) Total mean cells per follicle/slide under SPF housing ranged from 226–281. B-cells/total cells per follicle/slide peak at 24 weeks of age, whereas CD4+ and CD8+ T-cells and FDCs remain stable at all time points. (TIF) [file pone.0082355.s001.tif]
